# Comorbid Obsessive-Compulsive Symptoms in Schizophrenia - Diagnostic and Treatment Challenges

**DOI:** 10.1192/j.eurpsy.2023.2252

**Published:** 2023-07-19

**Authors:** J. A. Leal, J. C. Moura, T. C. Rocha, J. F. Cunha, S. P. Torres, D. Seabra, I. M. Lopes, M. E. Carneiro, S. M. Esteves, R. Cajão, G. Lima

**Affiliations:** 1 Psychiatry, Centro Hospitalar Barreiro Montijo, E.P.E., Setúbal, Portugal

## Abstract

**Introduction:**

The comorbidity between Schizophrenia and Obsessive-Compulsive Symptoms represents almost 25% of schizophrenic patients and it is believed that almost 12% match the diagnostic criteria for Obsessive-Compulsive Disorder. Some second-generation antipsychotics may worsen or even induce those symptoms, which makes the treatment of this patients a difficult challenge.

**Objectives:**

To assess the link between Schizophrenia and Obsessive-Compulsive Symptoms, to discuss the diagnostic challenges and treatment options. To present a clinical case report of a schizophrenic patient with Obsessive-Compulsive Symptoms, which improved with proper treatment.

**Methods:**

We performed a non-systematic review of the existent literature with the keywords “Schizophrenia” and “Obsessive-Compulsive Symptoms”. Description of a clinical case report.

**Results:**

We present the case report of a male, 21 years old, single, diagnosed with Schizophrenia. In the past year, he was admitted twice in a psychiatric ward for persecutory and mystic delusions, which lead him to erratic behaviour. Since his adolescence he manifested repeated washing and compulsive cleaning associated with the fear of being contaminated with multiple diseases. Those compulsions worsened when he started being treated with antipsychotics. However, with therapeutic adjustments and with the introduction of an antidepressant we were able to control those symptoms.

**Conclusions:**

Some antipsychotics may induce or even aggravate Obsessive-Compulsive Symptoms in psychotic patients. It is of extreme relevance to differentiate those symptoms as comorbid in Schizophrenia or if they existed prior to the first positive symptoms, since they can be representative of an Obsessive-Compulsive Disorder. Understanding this diagnostic and treatment complexity enables us to be more familiar with the development of Obsessive-Compulsive Symptoms in schizophrenic patients.

**Disclosure of Interest:**

None Declared

